# High Quality Long-Term CD4^+^ and CD8^+^ Effector Memory Populations Stimulated by DNA-LACK/MVA-LACK Regimen in *Leishmania major* BALB/c Model of Infection

**DOI:** 10.1371/journal.pone.0038859

**Published:** 2012-06-08

**Authors:** Lucas Sánchez-Sampedro, Carmen Elena Gómez, Ernesto Mejías-Pérez, Carlos Oscar S. Sorzano, Mariano Esteban

**Affiliations:** 1 Department of Molecular and Cellular Biology, Centro Nacional de Biotecnología, Consejo Superior de Investigaciones Científicas (CSIC), Madrid, Spain; 2 Biocomputing Unit, Centro Nacional de Biotecnología, Consejo Superior de Investigaciones Científicas (CSIC), Madrid, Spain; Federal University of São Paulo, Brazil

## Abstract

Heterologous vaccination based on priming with a plasmid DNA vector and boosting with an attenuated vaccinia virus MVA recombinant, with both vectors expressing the *Leishmania infantum* LACK antigen (DNA-LACK and MVA-LACK), has shown efficacy conferring protection in murine and canine models against cutaneus and visceral leishmaniasis, but the immune parameters of protection remain ill defined. Here we performed by flow cytometry an in depth analysis of the T cell populations induced in BALB/c mice during the vaccination protocol DNA-LACK/MVA-LACK, as well as after challenge with *L. major* parasites. In the adaptive response, there is a polyfunctional CD4^+^ and CD8^+^ T cell activation against LACK antigen. At the memory phase the heterologous vaccination induces high quality LACK-specific long-term CD4^+^ and CD8^+^ effector memory cells. After parasite challenge, there is a moderate boosting of LACK-specific CD4^+^ and CD8^+^ T cells. Anti-vector responses were largely CD8^+^-mediated. The immune parameters induced against LACK and triggered by the combined vaccination DNA/MVA protocol, like polyfunctionality of CD4^+^ and CD8^+^ T cells with an effector phenotype, could be relevant in protection against leishmaniasis.

## Introduction

Leishmaniasis is one of the most neglected tropical diseases, prevalent in 88 countries presenting an estimated annual incidence of 2 million infections and about 12 million cases worldwide [1]. There are few drugs for chemotherapy available and treatments are still long-lasting, highly toxic and expensive. The goal in chemotherapy still remains a safe cheap oral drug and this objective appears to be distant for both major forms of the disease [2]. All these evidences point out the development of an effective vaccine as a major need against leishmaniasis.

Several antigens and different vaccination procedures pursuing the development of a protective Th1 response against the parasite have been used in experimental vaccination trials in murine and canine leishmaniasis achieving varied protection levels [3]. Among all leishmania antigens used, studies comparing DNA vaccine candidates pointed out that one of the most promising genes is LACK [4]. LACK, the leishmania homologue for receptors of activated C kinase, is a 36 kDa intracellular protein that is expressed in both stages of the parasite (amastigote and promastigote) [5], is highly conserved among Leishmania species [6] and is also very immunogenic, being a preferential target for the early anti-parasite immune response. In the context of a natural infection, the early-activated LACK reactive cells exhibit a marked Th2 phenotype [7]. Some evidences pointed that this immune profile against LACK antigen can be altered, and this alteration is enough to induce resistance to infection [8].

T cells have a central function in protection against a broad range of pathogens. In particular, CD4^+^ and CD8^+^ T cells can be important in controlling disease development [9]. In the case of leishmaniasis, several studies have been performed to dissect the relevance of CD4^+^ and CD8^+^ T cell subsets and their relative role in natural infection [10], [11], [12], [13], [14], prophylaxis [15], [16], [17] or therapy [18]. However, due to the heterogeneity of T cell cytokine responses generated by different vaccines, there are still few defined immune correlates of protection for infections requiring T cell responses. Consequently, it is of a high importance to improve the understanding of functional heterogeneity of CD4^+^ and CD8^+^ T cell cytokine responses induced by the current vaccine candidates [15].

We have previously described that vaccination with DNA-LACK and MVA-LACK was able to confer protection against cutaneus leishmaniasis in BALB/c mice after challenging the animals with metacyclic promastigotes [19] and against visceral leishmaniasis in dogs [20]. In both cases protection was mediated by a Th1-like immune response against LACK antigen. However, a deep study of the immune populations involved in protection was still needed.

Multicolor Flow Citometry is a powerful tool to discriminate between different immune populations as it evaluates magnitude and quality of cellular responses [21]. In this work we analyzed by Intra Cellular Staining (ICS) the adaptive and memory T cell responses induced by prime/boost vaccination with DNA-LACK/MVA-LACK using markers that recognize T cell lineages (CD4, CD8), T cell functions (IFNγ, TNFα, IL-2) and memory stages (CD44, CD62L, CD127). In addition, we examined the impact of the challenge with purified metacyclic promastigotes in those T cell populations.

## Results

### DNA-LACK/MVA-LACK induces an adaptive antigen-specific T cell response mediated by CD4^+^ and CD8^+^ T cells with high polyfunctional profile

**Figure 1:**
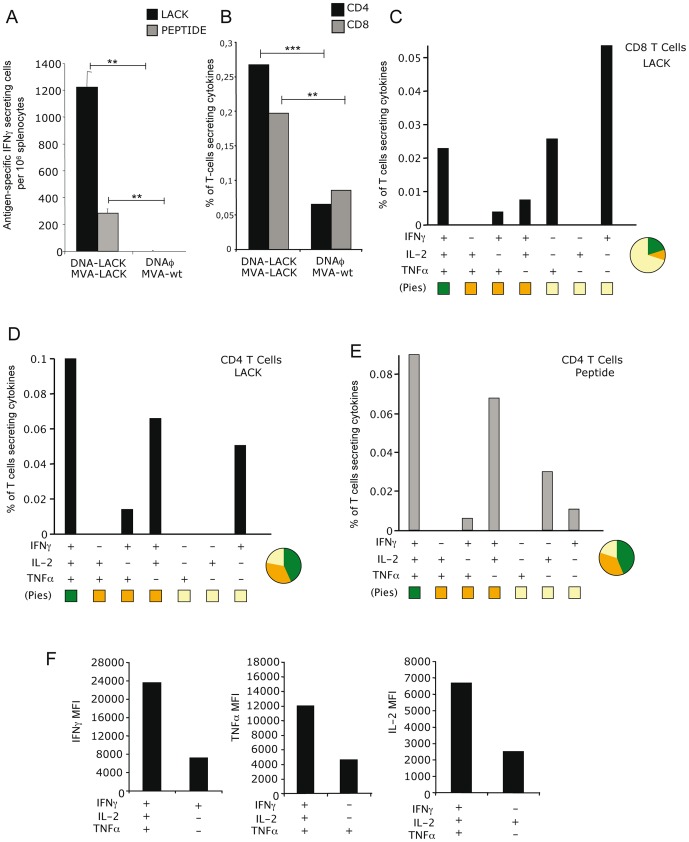
Adaptive immune response induced in mice immunized with the LACK vectors. A. Analysis of the antigen-specific IFNγ secreting cells by splenocytes measured by ELISPOT 11 days after boost. B. Analysis of the total magnitude of CD4^+^ and CD8^+^ T cell responses in splenocytes re-stimulated with LACK protein. Among the lymphocyte population, T cells were gated and analyzed for IFNγ, TNFα and/or IL-2 production. MVA-wt background was subtracted before representation. Cytokine production by LACK-specific CD8^+^ T cells (C), LACK-specific CD4^+^ T cells (D) or LACK_157–173_ peptide-specific CD4^+^ T cells (E). The different combinations of cytokines are indicated on the *x*-axis; percentages of T cells producing any cytokine are indicated on the *y*-axis. The different pies show the quality of the response measured as the relative quantity of single, double or triple cytokine producing cells (F). Analysis of the Geometric Mean of Fluorescence Intensity of IFNγ, TNFα or IL-2; produced by different populations of LACK-specific CD4^+^ T cells isolated from animals that received heterologous vaccination with DNA-LACK/MVA-LACK. Data is representative of two independent experiments.

We have previously described a heterologous prime/boost vaccination approach based on DNA and vaccinia virus vectors that induced protection against *L. major* infection in immunized BALB/c mice and this effect was Th1-dependent [19]. To analyze in more detail the vaccine-specific immune responses triggered in BALB/c mice by a DNA-LACK/MVA-LACK immunization regimen, groups of mice were first primed intradermally (i.d.) with 100 µg of DNA-LACK or sham DNA (DNAφ), and two weeks later the animals were boosted by intraperitoneal (i.p.) route with 2×10^7^ PFU/mouse of recombinant virus MVA-LACK or MVA-wt. Vaccine-elicited adaptive immune responses using splenocytes stimulated *in vitro* with either purified LACK protein or LACK_157–173_ peptide, were measured 11 days after the boost by fresh IFNγ ELISPOT and ICS assays. The number of IFNγ secreting cells detected against both stimulus was significantly increased in the group of animals that received DNA-LACK/MVA-LACK in comparison with control group (DNAφ/MVA-wt) (Fig. 1A).

To study the contribution of CD4^+^ and CD8^+^ T cell subsets in the overall LACK-specific immune responses, the sum of the frequency of antigen-specific T cells secreting IFNγ and/or IL-2 and/or TNFα was determined. At 11 days post-boost, there was a specific induction of CD4^+^ and CD8^+^ T cells in the group that received DNA-LACK/MVA-LACK in comparison with animals immunized with DNAφ/MVA-wt (Fig. 1B).

The simultaneous measurements of three cytokines allowed the assessment of the quality of the vaccine-induced CD4^+^ and CD8^+^ T cell responses. On the basis of the analysis of IFNγ, TNFα and IL-2 secretion, seven distinct LACK-specific CD4^+^ and CD8^+^ T cell populations were identified. As shown in Fig. 1C, the heterologous vaccination protocol DNA-LACK/MVA-LACK induces CD8^+^ T cells represented mainly by single positive cells secreting IFNγ or TNFα. However, around 78% of vaccine-induced CD4^+^ T cells showed high polyfunctional profile represented by T cells secreting three cytokines (43.19%), or two cytokines (34.81%) (Fig. 1D).

We also analyzed in more detail the phenotype of the adaptive immune response against LACK_157–173_, a crucial CD4^+^ T cell restricted peptide involved in the development of a Th2 response and susceptibility [22]. To this end, we evaluated the frequency of IFNγ, TNFα and/or IL-2 secreting cells after *in vitro* stimulation with LACK_157–173_ peptide. As expected, CD4^+^ T cells mediated the overall peptide-specific immune responses whereas no specific CD8^+^ T cell responses were detected. The vaccine-induced CD4^+^ T cell responses detected against LACK_157–173_ peptide at 11 days after the boost were highly polyfunctional with more than 75% of cells secreting 2 (36.12%) or 3 (43.78%) cytokines (Fig. 1E).

As it has been previously shown by others [15], there is a correlation between levels of secretion of cytokines and Fluorescence Intensity, thus we next measured the geometric Mean of Fluorescence Intensity (gMFI) within the different populations of LACK-specific CD4^+^ T cells. As a result, triple positive CD4^+^ T cells had a 3.28 fold increase in gMFI for IFNγ, 2.56 in the case of TNFα or 2.66 for IL-2 in comparison with single cytokine secreting cells (Fig. 1F).

The above findings showed that during adaptive immune responses the protocol DNA/MVA triggered CD4^+^ and CD8^+^ T cells that are polyfunctional.

### Effector memory CD4^+^ and CD8^+^ T cells with a polyfunctional profile are elicited by the heterologous DNA-LACK/MVA-LACK vaccination

**Figure 2:**
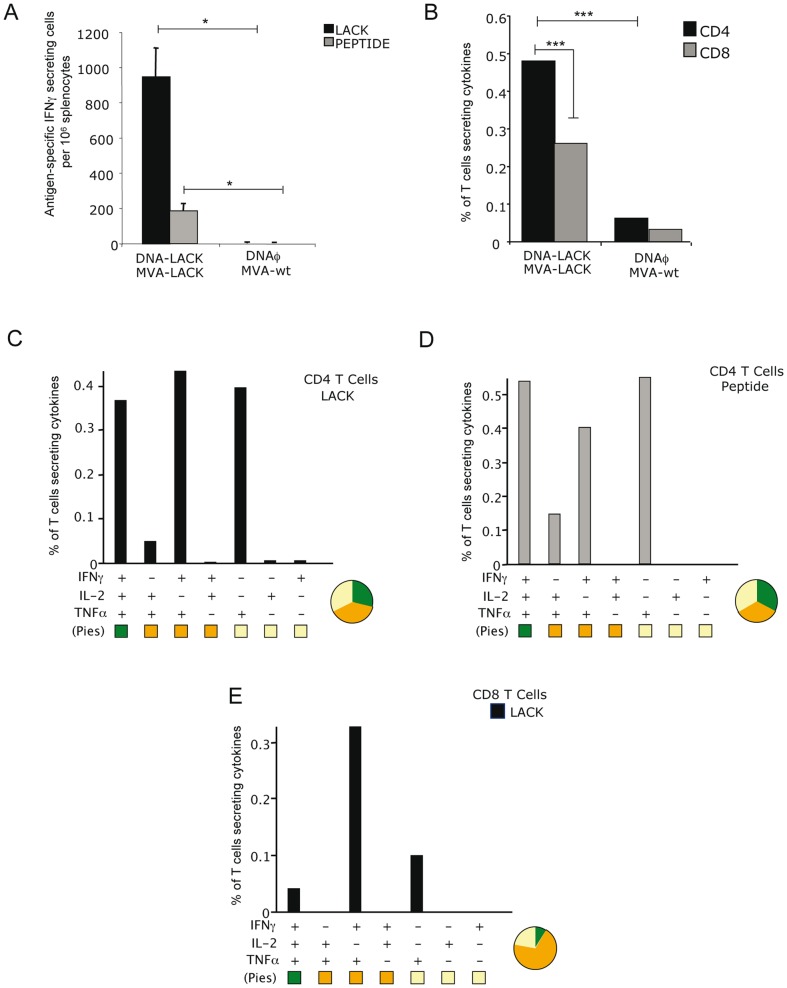
Memory immune response induced in mice immunized with the LACK vectors. A. Analysis of the antigen-specific IFNγ secreting cells by splenocytes measured by ELISPOT 53 days after the booster. B. Analysis of the total magnitude of effector memory (CD62L^−^ CD44^+^) LACK-specific CD4 and CD8^+^ T cell responses in splenocytes re-stimulated with LACK protein. Among the lymphocyte population, T cells were gated and analyzed for IFNγ, TNFα and/or IL-2 production. Cytokine production by effector memory (CD62L^−^ CD44^+^) LACK-specific CD8^+^ T cells (C), effector memory (CD62L^−^ CD44^+^) LACK-specific CD4^+^ T cells (D) and effector memory (CD62L^−^ CD44^+^) LACK_157–173_ peptide-specific CD4^+^ T cells (E). The different combinations of cytokines are indicated on the *x*-axis; percentages T cells producing any cytokine are indicated on the *y*-axis. The different pies show the quality of the response measured as the relative quantity of single, double or triple cytokine producing cells. Data is representative of two independent experiments.

Since memory T cell responses are critical to induce long-term protection against infection, we assessed at 53 days post-boost the memory profile elicited by DNA-LACK/MVA-LACK immunization. Vaccine-elicited memory immune responses in splenocytes from immunized animals stimulated with LACK protein or LACK_157–173_ peptide were first measured by IFNγ ELISPOT assay. As shown in Fig. 2A, DNA-LACK/MVA-LACK regimen induces strong and long-lasting antigen-specific IFNγ responses while in DNAφ/MVA-wt no antigen-specific responses were detected. The overall LACK-specific immune response at 53 days post-boost was mainly mediated by both CD4^+^ and CD8^+^ T cells (Fig. 2B).

To evaluate in more detail the phenotype of the LACK-specific memory T cells elicited by the immunization DNA/MVA protocol, splenocytes were stained for CD4, CD8, CD44 and CD62L surface markers in order to differentiate between the different memory populations. With those markers we can distinguish naïve (CD44− CD62L^+^), central memory (CD44^+^ CD62L^+^) and effector memory T cells (CD44^+^ CD62L^−^). We also evaluated by ICS IFNγ, TNFα and IL-2 secretion after *in vitro* stimulation with purified LACK protein or LACK_157–173_ peptide. Both CD4^+^ and CD8^+^ antigen-specific T cell responses have mainly an effector phenotype. As in the acute phase, at day 53 the heterologous vaccination induced CD4^+^ T cells that showed a high polyfunctional profile with T cells secreting three cytokines (29.10%) or different combinations of double positive secreting cells (38.46%) (Fig. 2C). Memory CD4^+^ T cells activated by the LACK_157–173_ peptide were also polyfunctional. More than 50% of the CD4^+^ T cells secreted two or more cytokines (33.73% of double positive or 32.82% of triple positive cells) (Fig. 2D). A major increase in TNFα single producers was observed in the specific-responses against both stimulus, being a 31,32% of the total CD4^+^ T cell response. The majority of the CD8^+^ T cell responses were represented by double cytokine producers (69.40%) while 21.72% were single TNFα producers (Fig. 2E).

In addition to CD62L and CD44 markers we considered staining the cells for CD62L and CD127 in order to determine whether effector cells at memory stage were indeed activated effector T cells (CD62L^−^CD127^−^) or effector memory T cells (CD62L^−^CD127^+^). We also evaluated by ICS IFNγ, TNFα and IL-2 secretion after *in vitro* stimulation with purified LACK protein or LACK_157–173_ peptide. As depicted in Fig S1 both CD4^+^ and CD8^+^ antigen-specific T cell responses have mainly a memory effector phenotype, with 81.8% of CD4^+^ and 61% of CD8^+^ of LACK-specific T cells secreting cytokines rare evealing a CD62L^−^CD127^+^ phenotype. Similar results (87.2%) were observed in CD4^+^ T cells re-stimulated with LACK_157–173_ peptide.

The above findings showed that, at the memory phase, both polyfunctional CD4^+^ and CD8^+^ T cells with an effector memory phenotype are activated by the DNA/MVA protocol.

### Enhanced CD4^+^ and CD8^+^ T cells are elicited by DNA-LACK/MVA-LACK vaccination at early stages after infection with metacyclic promastigotes of *L.major*

**Figure 3:**
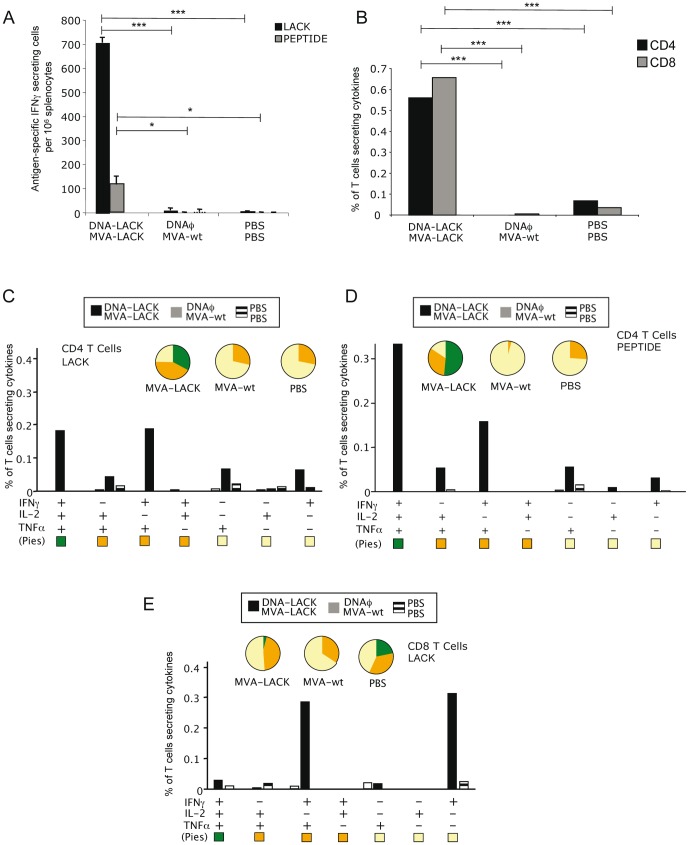
Immune response triggered in immunized mice after parasite challenge. A. Analysis of the antigen-specific IFNγ secreting cells by splenocytes measured by ELISPOT 10 days after the challenge. B. Analysis of the total magnitude of CD4^+^ and CD8^+^ T cell responses in splenocytes re-stimulated with LACK protein. Among the lymphocyte population, T cells were gated and analyzed for IFNγ, TNFα and/or IL-2 production. Cytokine production by LACK-specific CD4^+^ T cells (C), LACK_157–173_ peptide–specific CD4^+^ T cells (D), LACK_157–173_–specific CD8^+^ T cells (E). The different combinations of cytokines are indicated on the *x*-axis; percentages T cells producing any cytokine are indicated on the *y*-axis. The different pies show the quality of the response measured as the relative quantity of single, double or triple cytokine producing cells. Data is representative of two independent experiments.

To evaluate further the immune response to the combined DNA/MVA vaccine, we next determined the impact of a challenge infection on the immunological response in vaccinated mice. Thus, 3 groups of animals (4 animals per group) immunized with DNA-LACK/MVA-LACK, DNAφ/MVA-wt or PBS/PBS regimens, were infected with 5×10^4^ PNA purified *L.major* promastigotes 3 weeks after the booster, and 10 days after parasite challenge the animals were sacrificed and cellular immune responses were evaluated by ELISPOT and ICS. The parasite infection showed an ELISPOT profile of IFNγ secreting LACK-specific splenocytes (Fig. 3A) somewhat similar to that observed at pre-challenge (Fig. 1A). We next dissected the T cell responses by ICS. In animals vaccinated with DNA-LACK/MVA-LACK there is a significant increase in both CD8^+^ and CD4^+^ LACK-specific T cells frequencies measured as the sum of the frequency of antigen specific T cells secreting IFNγ and/or IL-2 and/or TNFα compared with the control groups (Fig. 3B). Both CD4^+^ and CD8^+^ T cell responses showed high polyfunctionality with 75.43% of CD4^+^ T cells (Fig. 3C) and 49.15% of CD8^+^ T cells producing 2 or 3 cytokines (Fig. 3E). CD4^+^ T cell (Fig. 3C) have a remarkable percentage of specific T cells secreting 3 cytokines (38.26%), or different combinations of double positive secreting cells (45.47%) when stimulated with LACK protein, and 51,48% of triple positive and 32% of double positive T cells when restimulated with LACK_157–173_ peptide (Fig. 3D). CD8^+^ LACK-specific T cell responses (Fig. 3E) were distributed in two different populations: IFNγ^+^/TNFα^+^ double positive cells (45.80%) or single IFNγ^+^ secreting cells (48,40%). Low or undetectable antigen-specific T cell responses were registered in DNAφ/MVA-wt or PBS/PBS control groups, indicating that the parasite did not elicit LACK-specific Th1 T cell responses. Splenocytes were also stimulated with LSA to assess overall T cell responses against the parasite. LSA-specific T cell responses were mainly represented by CD8^+^ T cells (Fig. S2A). Both CD8^+^ and CD4^+^ T-cell populations showed a low polyfunctional profile and were mainly represented by single IFNγ secreting cells (Fig. S2B and S2C).

The findings of Fig. 3 revealed that both CD4^+^ and CD8^+^ T cells with a polyfunctional profile are activated in DNA/MVA vaccinated animals after challenge with *L. major* at the acute phase after infection.

As it has been reported previously [23], the development of lesions in the foodpad of animals infected with *L. major* takes approximately 4 weeks. At that point vaccinated animals heal small swelling lesions and control animals develop cutaneous disease. To evaluate the state of the immunological response in vaccinated mice, 2 groups of animals (3 animals per group) receiving DNA-LACK/MVA-LACK or PBS/PBS, were infected with 5×10^4^ PNA purified *L. major* promastigotes 3 weeks after the booster, and 28 days after parasite challenge, the animals were sacrificed and cellular immune responses were evaluated by ELISPOT and ICS. The parasite infection showed an ELISPOT profile of LACK-specific IFNγ-secreting splenocytes (Fig. S3A) similar to that observed at pre-challenge (Fig. 1A), or 10 days postchallenge (Fig. 3A). Hence, the parasite challenge has little effect on boosting IFNγ response acquired by the heterologous DNA/MVA vaccination approach; notwithstanding this IFNγ response is maintained with time in infected animals.

We next dissected the T cell responses by ICS. In animals vaccinated with DNA-LACK/MVA-LACK there is an increase in LACK-specific CD8^+^ T cell frequencies measured as the sum of the frequency of antigen-specific T cells secreting IFNγ and/or IL-2 and/or TNFα (Fig. S3B). CD4^+^ T cell responses showed high polyfunctionality, with 74.9% of cells producing more than two cytokines; 37,47% were double and 37.43% triple positive (Fig. S3C). LACK-specific CD8^+^ T cell responses were distributed in two different populations: IFNγ^+^/TNFα^+^ double positive cells (25.98%) or single IFNγ^+^ secreting cells (58.75%) (Fig. S3D). Low or undetectable LACK-specific responses were registered in control groups. The findings of Fig. S3 revealed that polyfunctional CD4^+^ and CD8^+^ T cells activated in DNA/MVA vaccinated animals are maintained with time after challenge with *L. major*.

### Anti-vector immune responses in vaccinated mice are mainly polyfunctional CD8^+^ T cells

**Figure 4:**
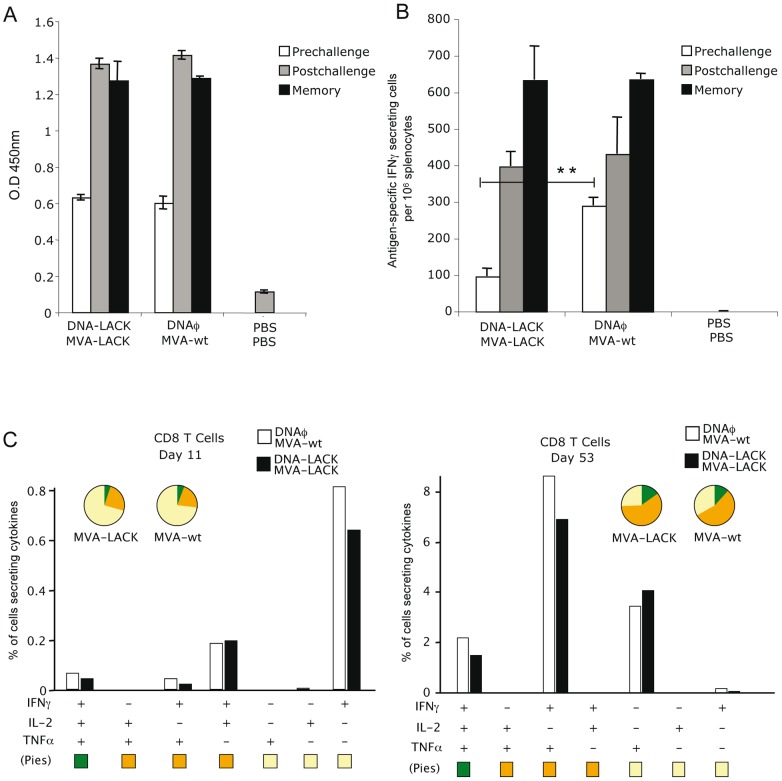
Analysis of humoral and cellular responses triggered by the MVA vector. A. IgG total antibodies at different points post-immunization where measured by ELISA using extracts of BSC40 cells infected with VACV. B. Analysis of the E3-specific IFNγ secreting cells by splenocytes measured by ELISPOT 11 days after the booster (prechallenge), 10 days after experimental challenge (postchallenge) or 53 days after the booster (memory). Among the lymphocyte population, T cells were gated and analyzed for IFNγ, TNFα and/or IL-2 production. C. Cytokine production by E3-specific CD8^+^ T cells 11 or 53 days after the booster.

To further characterize the immune responses induced in mice immunized with DNA-LACK/MVA-LACK, it was of interest to evaluate the responses against the MVA vector. Thus, we measured both antibodies and T cell responses in the group of mice immunized as in Figs. 1 and 2. IgG responses to vaccinia virus vector were observed in the group of animals vaccinated with DNA-LACK/MVA-LACK and DNAφ/MVA-wt at prechallenge, postchallenge and memory phases (Fig. 4A). Similar levels were observed at postchallenge and memory phases, indicating the stability of anti-vaccinia antibodies in the blood serum.

The cellular response to the vector was evaluated by ELISPOT after splenocyte stimulation with a vaccinia virus specific T cell peptide, corresponding to the virus E3 protein. The E3 peptide is one of the most immunogenic vaccinia peptides in BALB/c mice [24]. The main increase of E3-specific IFNγ T cells was observed at the memory phase (Fig. 4B). By ICS the majority of the anti-vector T cells were of CD8^+^ phenotype, with higher magnitude and polyfunctional profile at memory phase than at 11 days after booster (Fig. 4C).

The above findings showed that both humoral and T cell responses to VACV are induced by the DNA-LACK/MVA-LACK protocol and anti-vector CD8^+^ T cells are preferentially induced.

## Discussion

The LACK antigen is one of the most extensively studied proteins of Leishmania, because when it is administered as a DNA vector, as a protein or in combination with cytokines or in heterologous prime/boost protocols with viral vectors, induced protection against parasite challenge in mice and dogs [20], [25], [26]. Since it has been proposed that the quality of a CD4^+^ T cell cytokine response can be a crucial determinant in whether a vaccine is protective [15], we have defined in this investigation the immunological profile of cellular responses induced in heterologous DNA-LACK prime and MVA-LACK boost vaccination approach in mice. We have previously shown efficacy of this protocol against *L. major* in mice and dogs [19], [20] and protection levels in those studies were associated with an overall Th1 response against LACK antigen [19], [27]. Thus, vaccinia virus worked as a strong adjuvant and delivery vector altogether, modifying natural Th2-IL-4 response to LACK antigen dependent of Vβ4 Vα8 CD4^+^ T cells [7].

BALB/c mice develop a polarized and fatal Th2 immune response to subcutaneous *Leishmania major* infection due principally to the high immunogenicity of LACK antigen [5], in particular to the immunogenicity of the LACK_157–173_ CD4^+^-restricted peptide [22], [28], [29], [30]. Here we studied in detail the different T cell populations activated in BALB/c mice using the DNA/MVA vaccination approach, since Th1 responses driven mainly by CD4^+^ and CD8^+^ T cells have a major role in the immunology of cutaneus and visceral leishmaniasis [9]. We found that the adaptive immune response elicited by this protocol is characterized by a marked Th1 phenotype to the LACK antigen, represented by a low frequency but high quality CD4^+^ T cells against LACK antigen and its dominant LACK_157–173_ peptide, with more than 75% of those LACK-specific or LACK_157–173_ peptide-specific T cells expressing two or more cytokines. The importance of these multifunctional T cells might be explained by the fact that each single cell is capable of a broader repertoire of functions. These polyfunctional cells also showed an increase of the Mean Fluorescence Intensity (MFI) for IFNγ, TNFα and IL-2, meaning that the high quality profile of these cells also reflect higher levels of cytokine secretion on a per-cell basis. This last phenomenon was also observed in post-challenge and memory studies (not shown).

Moreover, we have seen that the parasite at early stages of the infection in vaccinated animals boosted both CD8^+^ and CD4^+^ T cells. LACK-specific CD8^+^ T cells producing altogether IFNγ and TNFα was observed. As differences in the profile of cytokines produced by individual cells have profound implications for their capacity to mediate effector function, this specific population is particularly relevant because both cytokines synergize in their capacity to mediate killing of leishmania through the induction of NO [31], [32]. This increase in CD8^+^ T cells is also noticed 4 weeks after experimental challenge with *L. major*. Although CD4^+^ T cells maintain magnitude and quality, there is an increment in CD8^+^ T cells magnitude represented in a 70% of cells secreting IFNγ.

We also observed that the resulting memory population 53 days after booster is mostly represented by high quality effector CD4^+^ T cells, with around 40% of CD44^+^ CD62L^−^ T cells secreting two or more cytokines.

From a vaccine point of view it was relevant to find at the memory stage an increase in the production of TNFα in both CD8^+^ and CD4^+^ LACK-specific effector memory T cells (Fig. 2), as TNFα has important stimulatory effects on macrophage killing activity [33], and its production has been shown to correlate with protection [34], [35]. It has been also demonstrated that CD4^+^ T cells that expresses IFNγ and TNFα play an important role in therapy against leishmaniasis [36]. It is important to point out that mice deficient in this cytokine failed in controlling cutaneus and visceral infection produced by Leishmania [37], [38].

We further confirm that the immune cells were of effector memory phenotype since the antigen-specific CD4^+^ and CD8^+^ T cell populations were mainly CD62L^−^CD127^+^. These results agree with recent publications that showed that in heterologous prime-boost vaccination regimen, CD8^+^ effector memory cells are the most relevant cells for long term protective immunity against parasites [39], [40], but also suggest that high quality effector memory CD4 T cells might play a relevant role in protection.

Darrah et all observed that immunization withvarious vaccine formulations with adenovirus vectors encoding specific *L. major* antigen (Leish-111f) or after primary infection with *L. major*, induced the expansion of multifunctional CD4^+^ T cells that secrete IFNγ, IL-2 and TNFα which strongly correlate with protection against subsequent challenge [15]. These authors also showed increased MFI for IFNγ and TNFα. Recently, it has been also reported the presence of this high quality, multifunctional CD4^+^ T cells in peripheral blood mononuclear cells of patients that healed American cutaneus leishmaniasis, and the same phenomenon was observed in the MFI for IFNγ, IL-2 and TNFα, thus supporting the significance of our findings [41]. In fact, in our DNA-LACK/MVA-LACK system we also observed multifunctional CD4+ T cells that secrete IFNγ, IL-2 and TNFα, but in addition we obtained CD8^+^ T cell responses which are also polyfunctional. The CD8^+^ T cells could be driven by the MVA vector, as the responses to a VACV antigen are mainly CD8^+^ T cells (Fig. 4C–D).

In conclusion, these results demonstrate that the expression of LACK protein in a DNA prime/MVA boost regimen can lead to the proliferation of a LACK-specific high quality subset of adaptive and memory CD4^+^ T cells with broad functionality that secrete a spectrum of Th1 cytokines. The heterologous vaccination also induced high quality LACK-specific CD4^+^ and CD8^+^ T cell effector memory populations. Anti-vector responses were largely CD8-mediated. The CD4^+^ T cell cytokine response to LACK is compatible with the findings that this T cell profile can be crucial in a protective response [42]. Thus, the immune parameters triggered by the combined vaccination DNA-LACK/MVA-LACK protocol are likely to be relevant in the protection conferred against leishmaniasis that we have previously described in mice and dogs using the same protocol.

## Materials and Methods

### Ethics Statement

The Ethical Committee of Animal Experimentation (CEEA-CNB) of Centro Nacional de Biotecnología (CNB-CSIC) approved the animal studies in accordance with national and international guidelines and with the Royal Decree (RD 1201/2005). Permit number: 080011.

### Parasite strains and animals

*L. major* (WHOM/IR/-173) was a kind gift from Dr Nicholas Glaichenhaus (CNSR, Valbonne, France). Promastigotes were cultured at 27°C in Schneider's medium (Gibco BRL, UK), supplemented with 20% fetal calf serum (FCS, Gibco BRL, UK) and antibiotics. The virulence of the strain was preserved by periodic passage through BALB/c mice. Frozen stocks grown in culture until the stationary phase were used for the experiments. In order to purify only infective metacyclic promastigotes stationary phase cultures were resuspended in endotoxin-free phosphate buffered saline (PBS) at a final concentration of 1–5×10^8^ promastigotes/ml and treated with 10 mg/ml of Peanut Agglutinin (PNA). After centrifugation at 1000 rpm, metacyclic promastigotes were collected from the supernatant, washed twice with PBS and resuspended in the same buffer for inoculation. All mice used in this study were female BALB/c mice ranging from 6 to 8 weeks of age purchased from Harlan (IN, USA) and housed in the Animal Facility of the Centro Nacional de Biotecnología-CSIC (Madrid, Spain) under pathogen-free conditions.

### Cells, plasmids and viruses

Cells were maintained in a humidified air 5% CO_2_ atmosphere at 37°C. Primary chicken embryo fibroblasts (CEF) were obtained from pathogen-free (SPF) 11-day-old eggs (Intervet, Salamanca, Spain) and were grown in Dulbecco's modified Eagle's medium (DMEM) supplemented with 10% FCS.

The mammalian expression plasmid vector pCI-neo-LACK, named here as DNA-LACK, was previously described [19]. The empty plasmid pCI-neo (Promega) was used as control (DNAφ). Both constructs were purified using the Qiagen plasmid purification kit (Qiagen).

The viruses used in this study included the highly attenuated Modified Vaccinia Ankara strain (MVA-wt, obtained at passage 586) or the recombinant MVA expressing the *Leishmania major* LACK antigen in the viral HA locus, lacking a selectable marker (MVA-LACK). All viruses were grown in CEF cells. Purification and titration of viruses was performed as previosly described [43], [44].

Plasmids and viruses were diluted for inoculation in endotoxin-free PBS.

### Reagents

*L. infantum* LACK protein was expressed as a fusion protein with a histidine tag at the N-terminal end using the *Escherichia coli* strain BL21 pLysS transformed with the plasmid pRSET-B-LACK [45], which allows its purification by affinity chromatography on a Ni^2+^ column. Bacteria were grown in the presence of antibiotic and the expression of the protein was induced by the addition of isopropyl thio-β-D-galactoside (IPTG) at a final concentration of 0.5 mM. Cultures were centrifuged (5000 rpm 15 min) and resuspended in Lysis Buffer (8 M urea, 50 mM Tris-HCL pH 8, 500 mM NaCl) and incubated 1 hour while being mixed in over-end in a tube rotator at 4°C. After 30 min centrifugation at 11000 rpm supernatant was incubated 1 hour while being mixed in over-end in a tube rotator at 4°C with 2 ml of equilibrated ProBond Nickel-Chelating Resin (Invitrogen). Beads were washed three times with Wash Buffer (8 M urea, 50 mM Tris-HCL pH 8, 500 mM NaCl, 10 mM imidazol) and elution was done increasing Imidazol final concentration up to 200 mM. PD10 Desalting Columns (GE Healthcare) were used to finally keep LACK protein in saline for *in vitro* studies. Protein preparations were analysed by Coomassie stain and Western-blot.

Leishmania soluble antigen (LSA) was prepared from late stationary phase *L. major* promastigotes. Cultures were collected and resuspended in 10 ml of phosphate buffer saline (PBS). After five freeze–thaw cycles, the suspension was centrifuged at 3000 rpm and the supernatant was collected in 1 ml aliquots. The protein concentrations were estimated using the BCA method (Pierce, Rockford, IL). The protein purity and concentration was determined by SDS-PAGE and BCA method, respectively (Pierce, Rockford, IL).

LACK_157–173_ peptide (FSPSLEHPIVVSGSWDN) was chemically synthesized at Proteomic Service, CNB.

LACK and LSA were sonicated during three cycles of 1 min each at 30% output power (Sonifier Cell Disrupter, Heat Systems Ultrasonic, Inc.) when used as stimulus in the ELISPOT and ICS assays.

### Immunization and infectious challenge

BALB/c mice, 6–8 weeks of age, were primed intradermally (i.d.) in the abdomen with 100 µg of DNA-LACK or DNAφ in 100 µl volume per mouse. At day 14 mice were boosted intraperitoneally (i.p.) with MVA-LACK or MVA-wt (2×10^7^ PFU/mouse) and PBS as control. Eleven days after boosting (day 25), three or five mice per group were sacrificed and sera and spleens were collected to analyze adaptive immune responses. Three weeks after boosting (day 46) five animals per group were challenged subcutaneously in the right hind footpad with 5×10^4^ metacyclic PNA-purified *L. major* promastigotes resuspended in 10 µl using BD Micro-Fine (BD Consumer Healthcare) 0,5 ml 30G needles. Mice were sacrificed 10 days post-challenge, and sera and spleens were harvested for immunological studies. For the memory studies, the remaining 5 vaccinated animals were sacrificed 53 days after boosting and sera and spleens were harvested for immunological studies. Two independent experiments were performed.

### Antibody measurement by ELISA

Presence of LACK-specific IgGs, IgG1 and IgG2a in serum samples, was assessed as previously described [26]. Serum from individual mice was reacted at 1∶100 dilution in triplicates against 10 µg/ml of extracts obtained from epithelial monkey kidney BSC40 cells infected with vaccinia virus (VACV) strain WR and treated with 0.5% NP40.

### Evaluation of specific T cells by the ELISPOT assay

The enzyme-linked immunospot (ELISPOT) assay to detect antigen-specific T cells was performed as described previously [26]. Briefly, 96-well nitrocellulose plates were coated with 6 µg/ml of anti-mouse IFNγ monoclonal antibody R4-6A2 (PharMingen, San Diego, CA) in 75 µl of PBS. Following overnight incubation at room temperature, the wells were washed three times with RPMI 1640 and blocked with complete medium supplemented with 10% FCS during 1 h at 37°C. Triplicate cultures of erythrocyte-depleted spleen cells were prepared from immunized mouse splenocytes in two dilutions, 10^6^ and 5×10^5^/well. Splenocytes were used as a source of antigen presenting cells. The number of IFNγ secreting cells specific for the different antigens was evaluated by pulsing the splenocytes with either, 25 µg/ml of sonicated recombinant LACK protein, 2 µg/ml of LACK_157–173_ peptide, 10 µg/ml of LSA or 10 µg/ml of E3 peptide. Plates were incubated for 18 h at 37°C with a 5% CO_2_ atmosphere, washed extensively with PBS–0.05% Tween (PBS-T) and incubated for 2 h at room temperature with 2 µg of biotinylated anti-mouse IFNγ monoclonal antibody XMG1.2 (PharMingen, San Diego, CA) per ml in PBS-T. Afterwards, plates were washed with PBS-T and 100 µl of peroxidase-labeled avidin (PharMingen, San Diego, CA) at a 1/800 dilution in PBS-T was added to each well. After 1 h of incubation at room temperature, wells were washed with PBS-T and PBS. The spots were developed by the addition of 3,3′-diaminobenzidine tetrahydrochloride substrate (1 mg/ml) (Sigma, St. Louis, MO) in 50 mM Tris–HCl, pH 7.5 containing 0.015% hydrogen peroxide and were counted with an AID ELISPOT reader system (Vitro).

### Intracellular Cytokine Staining assay (ICS)

The phenotypes of responding T cells were analyzed by ICS and fluorescence-activated cell sorting analysis as described elsewhere [46]. After an overnight rest, 5×10^6^ splenocytes (depleted of red blood cells) were stimulated with 25 µg/ml of LACK protein, 2 µg/ml of LACK_157–173_ peptide or 10 µg/ml of E3 peptide during 2 hours and left 4 hours in RPMI 1640 supplemented with 10% FCS and containing 1 µl/well Golgiplug (BD Biosciences) to inhibit cytokine secretion. Stimulation with LSA was performed overnight and as with the other stimulus splenocytes remained the last 4 hours in RPMI 1640 supplemented with 10% FCS and containing 1 µl/well Golgiplug (BD Biosciences) to inhibit cytokine secretion. After stimulation, cells were washed, stained for the surface markers, fixed, permeabilized using the BD Cytofix/Cytoperm™ Kit (Becton Dickinson) and stained intracellularly using the appropriate fluorochromes. To analyze the adaptive and post-challenge immune responses, the following fluorochrome-conjugated antibodies were used: CD3-FITC, CD4-Alexa 700, CD8-PerCP, IL-2-PE, IFNγ-APC and TNFα-PECy-7. For memory analyses, the following antibodies were used: CD4-Alexa 700, CD8-V500, CD62L-FITC, CD44-SPRD, CD127-PECy5.5, IFNγ-PECy-7, TNFα-PE and IL-2-APC. All antibodies were from BD Biosciences. Cells were acquired using an LSRII flow cytometer (Becton Dickinson) equipped with a high throughput system. The number of events ranged between 10^5^ and 10^6^. Dead cells were excluded using the violet LIVE/DEAD stain kit (Invitrogen). Lymphocytes were gated on a forward scatter area versus side scatter area pseudo-color dot plot. To analyze the adaptive immune responses, CD4^+^ and CD8^+^ events (gated previously on CD3^+^ cells) were gated versus IFNγ, TNFα and IL-2, and then combined together using the boolean operator. For memory analyses, CD4^+^ and CD8^+^ events were gated versus CD44 and CD62L or CD62L and CD127 to analyze the memory phenotype. IFNγ, TNFα and IL-2 were gated in the different memory populations and then combined together using the boolean operator. Sample analysis was performed using FlowJo version 8.5.3 (Tree Star, Ashland, OR).

### Statistical analysis

Statistical significance (p<0.05 (*), p<0.005 (**) or p<0.001 (***)) of differences between immunization groups of mice was determined by Student's t-test.

For the statistical analysis of ICS data we used a novel approach that corrects measurements for the medium response (RPMI) and at the same time allows the calculation of confidence intervals and p-values of hypothesis tests [47], [48].

The data analysis program, Simplified Presentation of Incredibly Complex Evaluations (SPICE, version 4.1.5, Mario Roederer, Vaccine Research Center, NIAID, NIH), was used to analyze and generate graphical representations of T cell responses detected by polychromatic flow citometry. All values used for analyzing proportionate representation of responses are background-subtracted.

## Supporting Information

Figure S1 **Analysis of the phenotype of memory antigen-specific CD4^+^ and CD8^+^ T cells in splenocytes re-stimulated with LACK protein or LACK peptide.** Memory T cells were classified as central memory (CD62L^+^CD127^+^), effector memory (CD62L^−^ CD127^+^) or effector (CD62L^−^ CD127^−^). Percentages represent the frequencies of T cells secreting IFNγ and/or TNFα and/or IL-2.
(TIF)

Figure S2 **Cytokine production by antigen-specific T cells 10 days after parasite challenge.** (A) Analysis of the total magnitude of CD4^+^ and CD8^+^ T cell responses in splenocytes re-stimulated with LSA. Among the lymphocyte population, T cells were gated and analyzed for IFNγ, TNFα and/or IL-2 production. Cytokine production by LSA-specific CD8^+^ T cells (B) or LSA-specific CD4^+^ T cells (C). The different combinations of cytokines are indicated on the *x*-axis; percentages of T cells producing any cytokine are indicated on the *y*-axis. The different pies show the quality of the response measured as the relative quantity of single, double or triple cytokine producing cells. Data is representative of two independent experiments.
(TIF)

Figure S3 **Immune profile 4 weeks after parasite challenge.** A. Analysis of the antigen-specific IFNγ secreting cells by splenocytes measured by ELISPOT. B. Analysis of the total magnitude of CD4^+^ and CD8^+^ T cell responses in splenocytes re-stimulated with LACK protein. Among the lymphocyte population, T cells were gated and analyzed for IFNγ, TNFα and IL-2 production. Cytokine production by LACK-specific CD4^+^ T cells (C) and LACK-specific CD8^+^ T cells (D). The different combinations of cytokines are indicated on the *x*-axis; percentages of T cells producing any cytokine are indicated on the *y*-axis. The different pies show the quality of the response measured as the relative quantity of single, double or triple cytokine producing cells.
(TIF)
