# Supplementary figures and images for: High Quality Long-Term CD4+ and CD8+ Effector Memory Populations Stimulated by DNA-LACK/MVA-LACK Regimen in Leishmania major BALB/c Model of Infection
Source: PLoS One. 2012 Jun 8;7(6):e38859. doi: 10.1371/journal.pone.0038859 (PMC3371028; doi:10.1371/journal.pone.0038859)

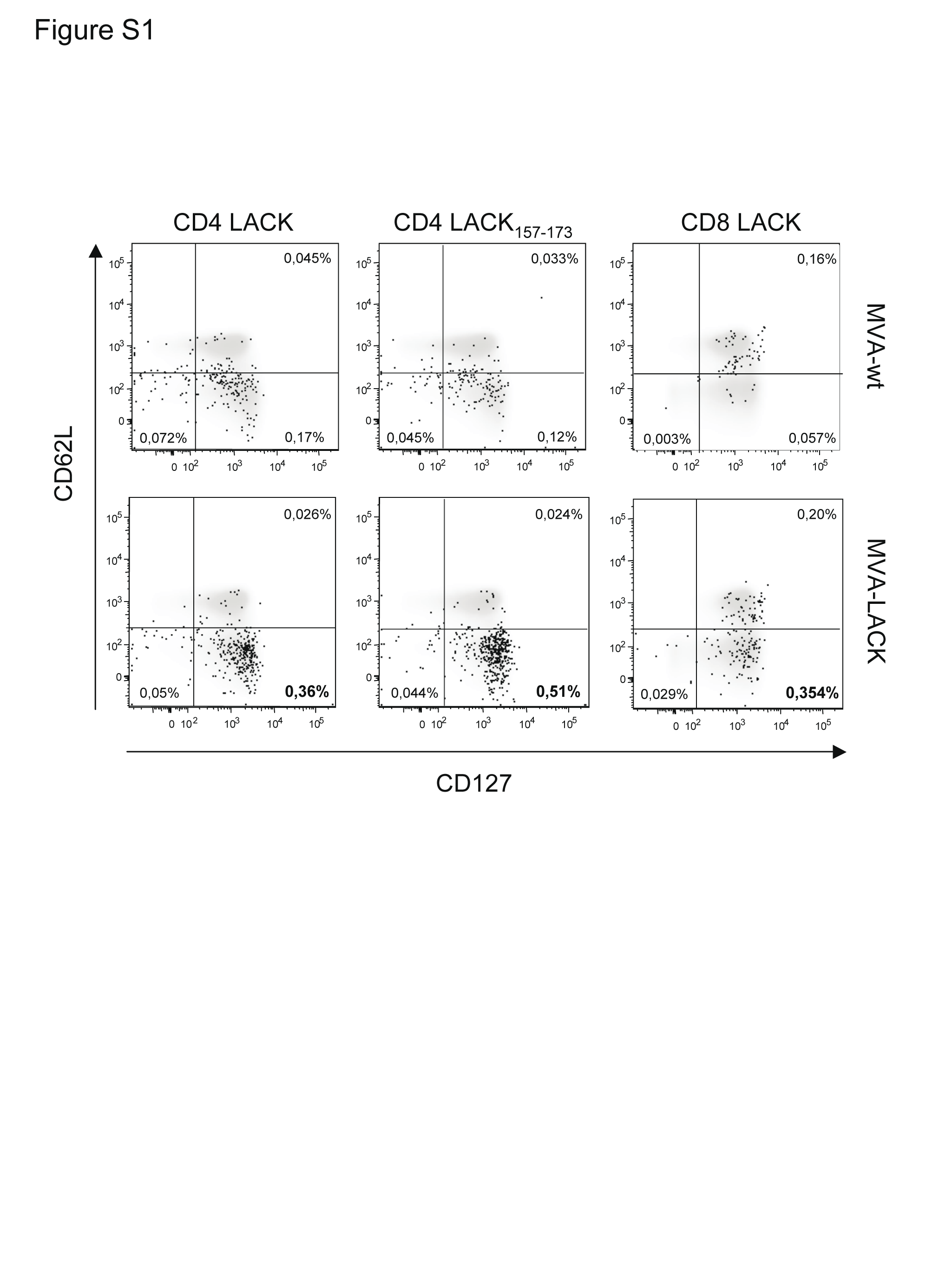

Supplement: Figure S1 — Analysis of the phenotype of memory antigen-specific CD4+ and CD8+ T cells in splenocytes re-stimulated with LACK protein or LACK peptide. Memory T cells were classified as central memory (CD62L+CD127+), effector memory (CD62L− CD127+) or effector (CD62L− CD127−). Percentages represent the frequencies of T cells secreting IFNγ and/or TNFα and/or IL-2. (TIF) [file pone.0038859.s001.tif]

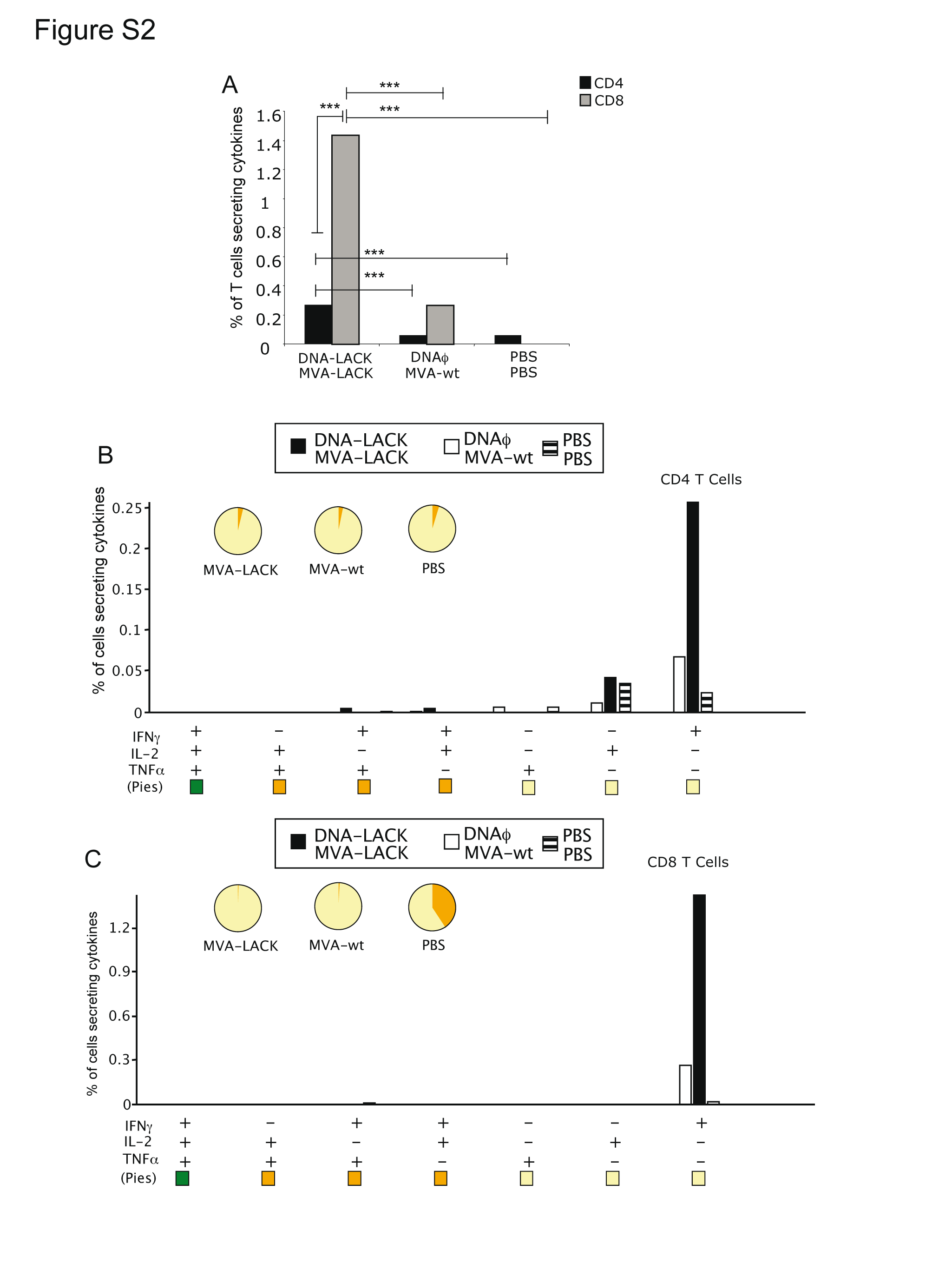

Supplement: Figure S2 — Cytokine production by antigen-specific T cells 10 days after parasite challenge. (A) Analysis of the total magnitude of CD4+ and CD8+ T cell responses in splenocytes re-stimulated with LSA. Among the lymphocyte population, T cells were gated and analyzed for IFNγ, TNFα and/or IL-2 production. Cytokine production by LSA-specific CD8+ T cells (B) or LSA-specific CD4+ T cells (C). The different combinations of cytokines are indicated on the x-axis; percentages of T cells producing any cytokine are indicated on the y-axis. The different pies show the quality of the response measured as the relative quantity of single, double or triple cytokine producing cells. Data is representative of two independent experiments. (TIF) [file pone.0038859.s002.tif]

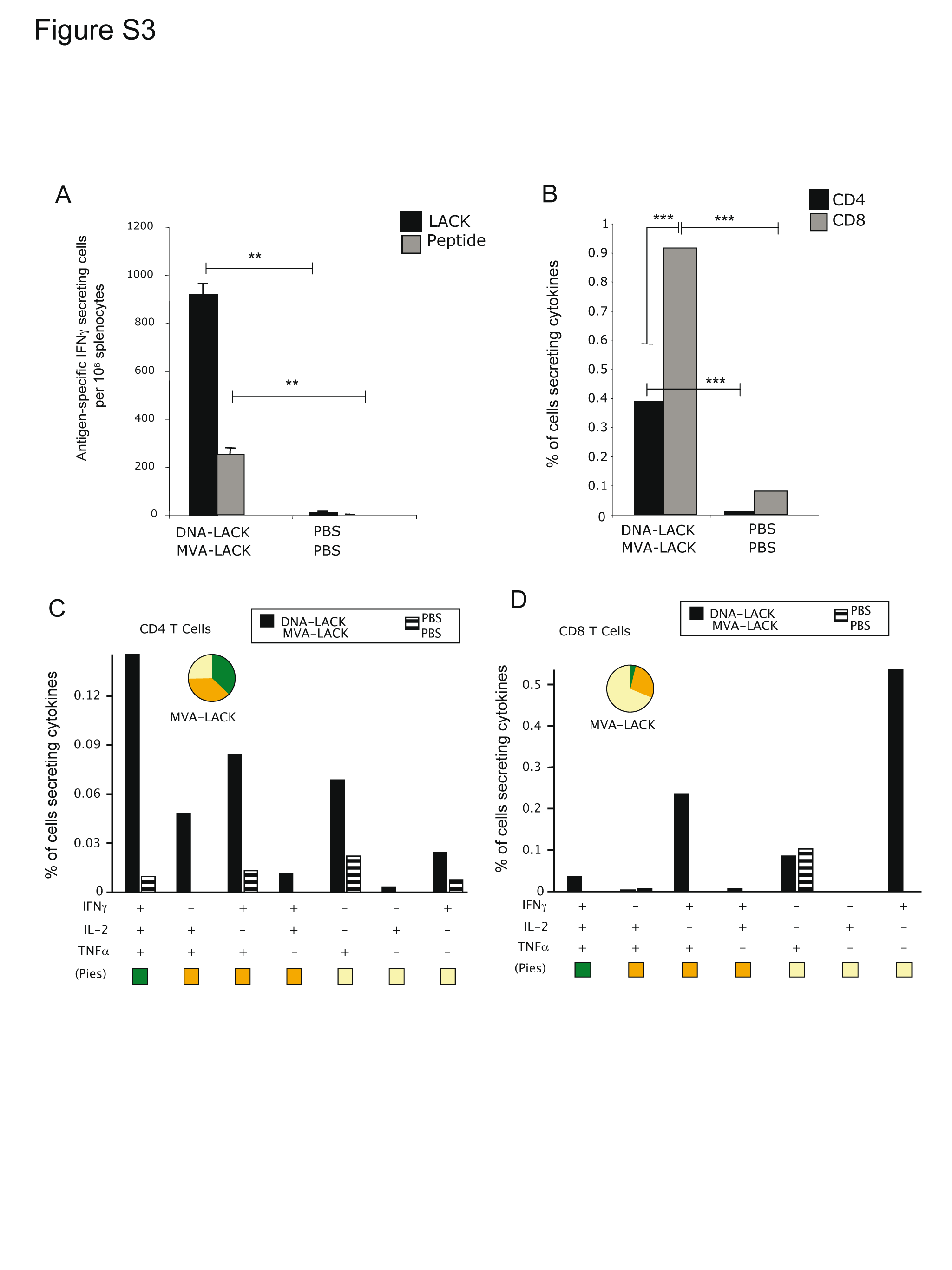

Supplement: Figure S3 — Immune profile 4 weeks after parasite challenge. A. Analysis of the antigen-specific IFNγ secreting cells by splenocytes measured by ELISPOT. B. Analysis of the total magnitude of CD4+ and CD8+ T cell responses in splenocytes re-stimulated with LACK protein. Among the lymphocyte population, T cells were gated and analyzed for IFNγ, TNFα and IL-2 production. Cytokine production by LACK-specific CD4+ T cells (C) and LACK-specific CD8+ T cells (D). The different combinations of cytokines are indicated on the x-axis; percentages of T cells producing any cytokine are indicated on the y-axis. The different pies show the quality of the response measured as the relative quantity of single, double or triple cytokine producing cells. (TIF) [file pone.0038859.s003.tif]
